# Basophils as Key Regulators of Allergic Inflammation and Th2-type Immunity

**DOI:** 10.1097/WOX.0b013e31817a76fb

**Published:** 2008-07-15

**Authors:** Bernhard F Gibbs

**Affiliations:** 1 Medway School of Pharmacy, University of Kent, Kent, UK

**Keywords:** basophils, mast cells, Th2 immunity, allergy

## Abstract

Basophils have long been suspected as playing more than a bystander role in initiating and maintaining allergic disorders, despite their relatively low numbers compared with other effector cells, such as mast cells and eosinophils. In vitro studies clearly demonstrated their propensity to generate proallergic cytokines, such as interleukin 4 and interleukin 13, as well as histamine and leukotrienes after simulation with allergens and innate IgE-dependent triggers. However, only very recently have mouse basophils been identified as key regulators of allergy in vivo, including orchestrating Th2 immunity to protease allergens in the induction phase. This review highlights these exciting advances that go far in unraveling our understanding of basophil function in the orchestration of allergic inflammation.

## 

Compared with other allergic effector cells, basophils are relatively rare and constitute only 1% or less of circulating leukocytes. Like their tissue-fixed mast cell counterparts, basophils express high-affinity immunoglobulin E (IgE) receptors (FcεRI), secrete histamine and eicosanoids (primarily LTC_4_) after IgE-mediated provocation, and undergo metachromatic staining. However, before the emergence of basophil-specific markers, it was hard to demonstrate their presence in organs affected by allergic inflammation. Despite this, in the early 1990s, indirect evidence pointed to their presence in these tissues during late-phase reactions based on their ability to release histamine without concomitant prostaglandin D_2 _(PGD_2_) or mast cell tryptase [1,2]. These observations increased interest in basophils as allergic effector cells, but during this period, few could have foreseen the crucial functions of these cells in actually orchestrating allergic inflammatory events, let alone possibly helper T cell type 2 (Th2) immunity itself.

A major turning point that changed our perceptions of allergic effector cells *per se *was the discovery that mast cells produce a variety of inflammatory and immunomodulatory cytokines. Murine mast cells or tumorigenic mast cell lines, in particular, were found to generate interleukin 1 (IL-1), IL-3, IL-4, IL-5, IL-6, IL-8, IL-13, granulocyte macrophage colony-stimulating factor, and tumor necrosis factor-α (TNF-α) besides several others after IgE-dependent activation[3] (reviewed in Gordon et al[4]). It was shown that mast cells could potentially drive developing helper T cell responses as well as up-regulate adhesion molecules on endothelial cells responsible for attracting other allergic inflammatory cells [5-7]. However, unlike rodent mast cells, the ability of primary human mast cells from either the lung or skin to generate Th2-type cytokines (especially IL-4) seems to be rather limited [8-11]. In contrast, human basophils rapidly synthesize and release IL-4,[12-15] in some cases even from preformed stores,[15] and IL-13,[15-17] cytokines that play a crucial role in supporting underlying atopy.

Although human basophils share certain characteristics with their mast cell counterparts, they differ in a number of important aspects regarding their ability to react to various stimuli and the types of mediators they release (Figure 1). In terms of cytokine synthesis, human basophils are relatively constrained to generating IL-4 and IL-13 rather than IL-5 or the multitude of proinflammatory cytokines ascribed to mast cells [18]. This sets the scene for the emergence of the basophil as a potential supporter of Th2 responses, evidence of which is highlighted in this review.

**Figure 1:**
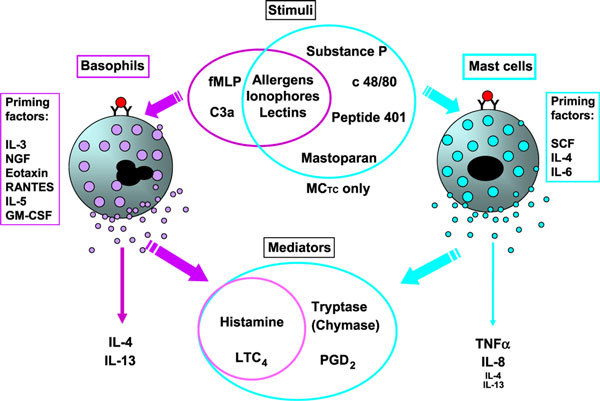
**Summary of human mast cell and basophil heterogeneity to stimulation and mediator generation (for better clarity, this is not a comprehensive list)**. Although both cell types react to IgE-mediated triggers, ionophores, basophils are more reactive to fMLP and C3a than most human mast cell types. Conversely, connective tissue mast cells (MC_TC_), but not mucosal mast cells (MC_T_) or basophils, are stimulated by neuropeptides and polybasic amines. In terms of mediator secretion, both cells secrete histamine and LTC_4_. Mast cells additionally release tryptase as well as, in the case of MCTC, chymase and PGs (primarily PGD_2_). Human basophils and mast cells differ in terms of cytokine synthesis: basophils are more restricted to IL-4 and IL-13 generation, whereas isolated mast cells produce TNF-a and IL-8 in vitro, although this may not reflect in vivo settings. Basophils are also known to respond to a large number of growth factors and chemokines that dramatically enhance their ability to generate mediators after IgE-dependent triggering. Human mast cells, on the other hand, are generally only responsive to priming by stem cell factor (SCF) and, to some extent, by IL-4 and IL-6. GM-CSF indicates granulocyte macrophage colony-stimulating factor; RANTES, regulated on activation normally T-cell expressed and secreted.

## Basophils are Major Sources of IL-4 and IL-13 and are Recruited to Tissues affected by Allergic Inflammation

Basophils express a wide variety of chemokine receptors (eg, CCR1-3, CXCR1, CXCR3, and CXCR4) and also respond to a range of chemokines and cytokines that facilitate their migration [19,20]. In recent years, the basophil-specific BB1 and 2D7 antibodies have been used with great success to demonstrate the presence of basophils in various tissues affected by allergic disease, such as the lung, skin, and nose (reviewed in Falcone et al[21]). During allergen-induced asthmatic reactions, basophils were shown to be responsible for 72% of IL-4 protein in the bronchial mucosa,[22] an observation that also correlates to murine asthma models [23]. In another study, Devouassoux and coworkers[24] demonstrated that the early production of IL-4 and IL-13 in the peripheral blood of asthmatic patients after allergen challenge was exclusively basophil derived. A real-time quantitative polymerase chain reaction-based assay in whole blood also identifies basophils as the most prominent source of IL-4 and IL-13 after stimulation with the cat allergen Fel d1 [25]. These findings verified the earlier in vitro investigations (described previously) showing that basophils are geared to rapid generation of these cytokines.

Interleukin 4 and IL-13 are archetypal proallergic cytokines. They increase vascular cell adhesion molecule-1 expression on the microvascular endothelium and eotaxin synthesis from airway epithelial cells, supporting leukocyte influx into affected tissues during late-phase responses [26-28]. They also play a key role in B-cell immunoglobulin class switching to favor IgE synthesis, and IL-4 is a vital factor for the early differentiation of CD4^+ ^lymphocytes to a Th2 type (reviewed in Haas et al[29]).

**Figure 2:**
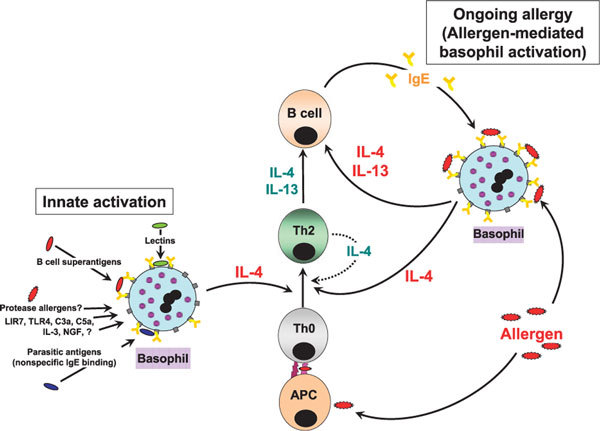
**Simplified overview of the immunomodulatory roles of basophils**. APC indicates antigen-presenting cells.

Given that basophils are major producers of these cytokines, an immunomodulatory role in supporting the previously mentioned events by these cells seems highly plausible (Figure 2). However, direct in vivo evidence for this has been, until very recently, scarce, owing largely to a lack of basophil knockout animal models because a basophil-specific growth factor has not yet been identified. Although growth factors such as IL-3, in particular, promote basophil development from CD34^+ ^progenitors (and controversially, murine mast cells, but not human mast cells), basophils are still present in IL-3-deficient mice [30]. This has stifled the development of basophil-knockout models analogous to mast cell-deficient mice (lacking c-kit receptors or c-kit ligand/stem cell factor, mandatory for mast cell maturation). To circumvent this problem, basophil-specific monoclonal antibodies have been used to deplete circulating murine basophils,[31] a technique that could potentially revolutionize future investigations into unraveling their biological roles.

## Basophils Regulate Late-Phase Reactions and Chronic Allergic Inflammation

Although murine mast cells can readily produce the Th2-type cytokines ascribed to human basophils, implying a redundancy in function for the latter, it is remarkable that some of the strongest evidence for a major role of basophils in allergic inflammation comes from murine allergy models. One such model uses transgenic mice generating 2,4,6-trinitrophenol (TNP)-specific IgE that gives rise to a variety of allergic responses after subcutaneous TNP injection [32]. This consists of early- and late-phase allergic responses, chronic allergic inflammation, and anaphylaxis after intravenous TNP injection. It was demonstrated that these mice fail to display signs of chronic allergic inflammation after prior depletion of circulating basophils [31]. Furthermore, a combinational approach, using a range of mice genetically defective in various lymphocytes and mast cells and reconstitution experiments, showed that mast cells, T cells, and natural killer cells (NK) and natural killer T cells (NKT) were not necessary for the late-phase allergic reactions, in stark contrast to basophils [33].

## Basophils as Potential Modulators Of Local IgE Synthesis

Recently, it has been shown that B cells residing in the bronchial mucosa of asthmatic patients undergo class switching to IgE synthesis, supporting earlier observations regarding their local generation of allergen-specific IgE in the nasal mucosa [34]. Gould et al[35] calculated that this local IgE production may account for a substantial portion of IgE saturating allergic effector cells within these sites. The fact that IgE synthesis occurs not only in the lymph nodes but locally within organs affected by allergic inflammation permits immunomodulatory input from resident allergic effector cells such as basophils.

Immunoglobulin E class switching is controlled by IL-4, IL-13, and CD40 ligand (CD40L) binding to its counterpart receptor on B cells. Yanagihara et al[36] showed that basophils can indeed direct polyclonal IgE synthesis in vitro by virtue of their ability to produce the previously mentioned stimuli, the effects of which were inhibited by the addition of neutralizing antibodies against IL-4, IL-13, and CD40L. In sharp contrast, cord blood-derived human mast cells failed to elicit this response. This is in agreement with an earlier study showing that although basophils (either primary or KU812 cell lines) and human mast cells (HMC-1) expressed CD40L, mast cells could only stimulate B-cell IgE synthesis in the presence of exogenously applied IL-4 [37]. Although these observations have yet to be fully verified by in vivo investigations, the liaison between tissue-associated basophils and B cells certainly represents a plausible *circulus vitiosus *of increasing IgE and effector cell sensitization central to the pathophysiology of allergic disease.

## Do Basophils Control Th2 Immunity?

As already discussed, it is increasingly clear that basophils are the main early source of IL-4 after allergen exposure in peripheral blood and asthmatic airways. Unlike IL-13, IL-4 is unique in driving CD4^+ ^T cells to a Th2 phenotype, which subsequently produce IL-4, IL-13, and other Th2-type cytokines themselves (reviewed in Haas et al[29]). The importance of IL-4 in initiating Th2 development was confirmed by studies showing that mice lacking either IL-4Rα or Stat6 (which controls IL-4-directed signaling) do not generate Th2 immune responses [38,39]. In addition to governing chronic allergic inflammation and perhaps also IgE synthesis, basophil-derived IL-4 may thus play a role in controlling Th2 immunity too. At the very least, basophils may exacerbate existing Th2 polarization in ongoing allergic disease. Further evidence for an association between basophils and Th2 lymphocytes is given by clinical studies showing that specific allergen immunotherapy reduces basophil reactivity and numbers as well as Th2 immunity [40].

Although there is strong evidence to show that basophils are capable of supporting local Th2 immune responses at sites of allergic inflammation and possibly regulating local B-cell IgE class switching, several important issues remain. First, do basophils migrate to the lymph nodes, which are the prime tissue sites for developing immune responses? Second, do basophils participate in the induction phase of Th2 immunity? The latter would require basophils to be activated by mechanisms that do not require the presence of surface-bound antigen-specific IgE.

Basophils are not usually attributed to being present in the lymph nodes. However, a recent study has clearly demonstrated that they transiently migrate into the draining lymph nodes of mice after immunization with the protease allergen, papain [41]. Interestingly, these investigations also showed that basophils were activated by protease allergen by innate triggering and released Th2 cytokines that were essential for initiating Th2 differentiation. A considerable proportion of activated basophils within the lymph nodes also released thymic stromal lymphopoietin (TSLP), in addition to IL-4, and were the only cell type within the lymph nodes that produced this Th2-driving factor. The TSLP-neutralizing antibodies indeed diminished Th2 differentiation without affecting either dendritic cell maturation and migration or basophil accumulation within the draining lymph nodes. This report clearly suggests that mouse basophils play a vital role in innate immune recognition leading to Th2 responses by secreting IL-4 and TSLP.

Although the previously mentioned data add further credence to the immunomodulatory capabilities of basophils, there are a number of unresolved issues. For example, TSLP production in human basophils has not yet been reported, and there is contradictory evidence regarding their ability to react to specific protease-activated receptor agonists[42] versus protease allergens (eg, Der p1) [43]. Elucidating the mechanisms of basophil migration into the lymph nodes after protease allergen challenge and whether this migration takes place in other settings also need addressing. For instance, although basophils have been shown to be critical in recruiting Th2 cells into affected tissues after *Nippostrongylus brasiliensis *infection (which also causes a substantial Th2-type response),[44] their innate activation was not necessary for Th2 differentiation in the lymph nodes [45]. Thus, although the evidence for basophil-directed immunomodulation within organs such as the lung is strong, their ability to control Th2 immunity, as well as IgE class switching, within lymphatic tissues is by no means fully resolved. This applies both to their ability to participate in early (innate) events of conditioning Th2 responses and to ongoing allergic disease, where their role in the lymphatic tissues is also not known.

## Innate Activators of basophil cytokine synthesis

There is now a wealth of literature showing that human basophils produce IL-4 and IL-13 in nonsensitized individuals by various innate triggers, some of which cause activation of FcεRI by binding to IgE in a non-antigen-specific manner (reviewed in Falcone et al[21,46]). These include parasitic antigens (eg, *Schistosoma mansoni, Echinococcus multilocularis*), lectins (eg, concanavalin A), and viral superantigens (eg, human immunodeficiency virus 1 gp120) that activate basophils by binding to non-antigen-specific IgE antibodies. Furthermore, basophil cytokine release is also caused by engagement of certain leukocyte immunoglobulinlike receptors (eg, LIR7), toll-like receptors (eg, TLR2), and to a more limited extent, by C5a/C3a and formyl-methionyl-leucyl-phenylalanine (fMLP).

In addition to the previously mentioned innate stimuli, basophil growth factors such as IL-3 and nerve growth factor (NGF) can also induce considerable levels of Th2-type cytokine synthesis [47,48]. Although this usually occurs only at relatively high concentrations of either IL-3 or NGF (above 10 ng/mL), lower amounts (1 ng/mL) substantially enhance IgE-dependent basophil mediator release to both specific allergens and innate activators. Regarding NGF, there is compelling evidence to suggest that this neurotrophin participates in both animal models of allergy and in human allergic asthma, where it correlates with disease severity and IgE levels [49,50]. Because basophils from asthmatic donors display a hyperreleaser phenotype,[51] factors such as NGF may have an important function in enhancing the synthesis of basophil-derived Th2-type cytokines and thereby their proallergic immunomodulatory roles (Figure 2).

## Basophils as Targets for Antiallergic Therapy

Given their potential in supporting both allergic inflammation and underlying Th2 immunity, basophils are an attractive target for antiallergic therapy. Their abilities to release mediators are inhibited by certain existing antiallergic agents such as glucocorticoids, methylxanthines, and calcineurin inhibitors, and this may partially explain their clinical effectiveness. Other traditional therapeutic agents, such as cromoglycate, have no known effects on basophils and highly variable actions on various mast cell subtypes.

The well-known limitations of these agents, including their heterogeneous actions on basophils and mast cells, have prompted renewed attempts to block allergen-mediated FcεRI activation of these cells. One promising approach is to prevent free IgE antibodies from binding to FcεRI using monoclonal anti-IgE (omalizumab), which reduces both effector cell sensitization and FcεRI expressions. Treatment with omalizumab in allergic rhinitis patients has been shown to strikingly reduce basophil reactivity and lead to a rapid decline in FcεRI expression, which was faster in comparison to mast cells [52]. In parallel, these authors also observed a reduction in allergen induced wheal size. A similar decline in basophil reactivity after omalizumab therapy is seen in allergic asthma, although in these settings, this was not always matched by clinical improvement [53].

Basophils may also be directly affected by rush desensitization regimens, which has been ascribed to a loss of Syk expression,[54] a crucial stimulatory signal associated with FcεRI activation. But their signal transduction machinery additionally encompasses a range of inhibitory signals that may contribute to their functional desensitization. One such signaling target is the *src *homology 2 domain-containing inositol 5ôphosphatase (SHIP), an inhibitory enzyme that is centrally involved in IgE-mediated signaling in both basophils and mast cells [55-57]. The SHIP has been shown to be mobilized upon the activation of inhibitory receptors, such as IRp60 (CD300a), leading to a dramatic reduction in allergic effector cell function [58]. CD300a, CD200R,[59] Siglec-8,[60] and FcγRIIb (the latter only blocks basophil responses in conjunction with FcεRI co-cross-linking[61]) up-regulate SHIP expressions and may in the future be exploited for antiallergic therapies.

## Conclusion

Recent in vivo investigations have provided exciting new inroads that support earlier in vitro studies showing that basophils are far from mere bystander cells of allergy and Th2 immunity. Their propensity to rapidly deliver Th2 cytokines under a variety of conditions and their necessity in eliciting chronic allergic inflammation underlines their immunomodulatory potential. However, the details concerning these actions still need to be elucidated, also in view of input from regulatory T cells that may down-regulate proallergic responses after parasitic helminth infections. The physiological purpose of basophils also remains elusive, as well as their interactions with other effector cells of the allergic inflammatory response. Nonetheless, there is overwhelming evidence to show that basophils contribute to the symptoms of allergic disease and support underlying Th2 immunity, especially in ongoing allergy. This cell therefore poses an obvious therapeutic target, and identifying ligands for inhibitory receptor engagement may be a promising approach to generate new antiallergic therapies.
